# Photoconversion efficiency of In_2_S_3_/ZnO core-shell heterostructures nanorod arrays deposited via controlled SILAR cycles

**DOI:** 10.1016/j.heliyon.2022.e09959

**Published:** 2022-07-16

**Authors:** Mohammed Rashid Almamari, Naser M. Ahmed, Araa Mebdir Holi, F.K. Yam, Mohammed Z. Al-Abri, M.A. Almessiere, Basma A. El-Badry, M.A. Ibrahem, Osamah A. Aldaghri, Khalid Hassan Ibnaouf

**Affiliations:** a School of Physics, Universiti Sains Malaysia, 11800 Penang, Malaysia; b Department of Medical Instrumentation Engineering, Dijlah University College, Baghdad, Iraq; c Department of Physics, College of Education, University of Al-Qadisiyah, Al-Diwaniyah, Al-Qadisiyah, 58002, Iraq; d Nanotechnology Research Center, Sultan Qaboos University, P.O Box 17, Al Khoud, Muscat, PC 123, Oman; e Department of Petroleum and Chemical Engineering, College of Engineering, Sultan Qaboos University, P.O Box 33, Al Khould, Muscat, PC 123, Oman; f Department of Physics, College of Science, Imam Abdulrahman Bin Faisal University, P.O BOX 1982, Dammam 31441, Saudi Arabia; g Department of Biophysics, Institute for Research & Medical Consultations (IRMC), Imam Abdulrahman Bin Faisal University, P.O. Box 1982, 31441, Dammam, Saudi Arabia; h Department of Physics, College of Science, Imam Mohammad Ibn Saud Islamic University (IMSIU), Riyadh 13318, Saudi Arabia; i Department of Physics, College of Women for Arts, Science and Education, Ain-Shams University, Cairo 11757, Egypt

**Keywords:** PCE, IZCSHNRAs, ZNRAs, ISNPs, SILAR cycles, Photoconversion and PECs

## Abstract

This paper reports the structures, morphologies, optical properties, and photoconversion efficiency (η%) of the In_2_S_3_/ZnO core-shell heterostructures nanorod arrays (IZCSHNRAs) produced via the controlled successive ionic layer absorption and reaction (SILAR) cycles. As-produced samples were characterized using XRD, FESEM, TEM, UV-Vis, PL, XPS and FTIR techniques. The proposed IZCSHNRAs revealed nearly double photocurrent density and η% values compared to the pure ZnO nanorod arrays (ZNRAs). In addition, the light absorption, crystallinity and microstructures of the specimens were appreciably improved with the increase of the SILAR cycles. The deposited nanoparticles of In_2_S_3_ (ISNPs) on the ZNRAs surface was responsible for the improvement in the heterostructures, light absorption and photogenerated electron–hole pairs separation, thus enhancing the photoconversion performance. It is established that a simple SILAR approach can be very useful to produce good quality IZCSHNRAs-based photoelectrodes required for the future development of high performance photoelectrochemical cells (PECs).

## Highlights

• In_2_S_3_/ZnO Heterostructures were produced via SILAR to be employed as PEC electrodes to enhance photoconversion efficiency.
• The effect of the various SILAR cycles on the morphologies, optics, and photoconversion of proposed IZCSHNRAs was assessed.
• The Type-II band was aligned using the ZnO and In_2_S_3_ band, enabling effective carrier separation and efficient transmission.
• High-quality, low-cost IZCHNRAs were performed using the SILAR with low InCl_3_ (cationic) and Na_2_S (anionic) concentrations.

## 1 Introduction

Currently, one of the main research goals worldwide is to develop a sustainable energy economy for the future development. Rapid depletion of the fossil fuel sources and technology expansion to meet the future energy demand posed an irrevocable disastrous effect on the ecosystem and cost of the fuels. Thus, it became essential to explore various new technologies in order to build an environmental friendly and cost-effective solution for the development of a sustainable energy economy [1, 2]. Novel technologies are required to maintain the projected energy consumption rate that is expected to be matchless. In this regard, the photoelectrochemical cell (PEC) is one of the most promising among all the developed technologies for providing the cleanest, cheapest, and locally produced energy. Such technology harvests a fraction of the solar radiation striking on the surface of Earth [3]. Many researchers have been enticed by the investion of Fujishima and Honda, first display of the PEC in 1972. Since then, numerous investigations have been made to achieve high performance PECs [4].

Khaslev and Turner [5] demonstrated a complicated monolithic PEC device that can successfully collect the solar energy and perform photoelectrolysis of water with the photoconversion efficiency (PCE) of 12.4%. Various researchers in the recent years have been focused to develop some cost-effective photoelectrodes for efficient performance of the PECs. The photoelectrodes are the heart of the PECs that must meet a complex set of parameters in order to achieve efficient photoconversion performance [6]. In short, a functional photoelectrode material must have the proper band characteristics, high quantum efficiency, and long-term anticorrosive properties [7]. The “holy grail in the semiconductor photo-electrochemistry” [8] refers to the search for a high performance semiconductor material. Although a broad array of semiconductor materials such as metal oxides and metal sulfides have been studied, the exploration for the perfect semiconductor still continues [9]. The metal oxide semiconductors (MOSCs) due to their high stability and low-cost are considered as the most promising PEC materials [10]. Conversely, the MOSCs have lower carrier mobility than silicon and other III-V semiconductors. Presently, the most difficult task is to overcome the limitations of the metal oxides while taking the advantages of their high stability and low-cost [11].

The applications of the photoelectrode materials for photoelectrochemical cells have increasingly focused on various nanomaterials [12, 13]. Different types of the MOSCs (like TiO_2_, ZnO, and WO_3_) are frequently employed as photoelectrodes in the PECs for the water splitting and hydrogen production due to their inexpensiveness, high stability and non-toxicity [14, 15, 16]. Amongst all the MOSCs, the ZnO nanorods (ZNRs) are preferred as electrode in the PECs for water splitting due to their desirable morphology, wide energy band gap and ease of fabrication [17]. Conversely, the PCE of the pure ZNRs for the photoelectrochemical water splitting applications is limited due to their optical absorption only in the ultraviolet (UV) region. Despite intensive studies a very high value of the optical absorbance of the ZNRs over a wide wavelength range is far from being achieved.

Several strategies have been adopted to improve the optical absorbance and PCE of ZnO which include nanostructure optimization [18], doping [19], sensitization [20], and so forth [21]. For improving the solar light usage and enabling the photon-generated carriers separation, fabrication of the semiconductor heterojunctions with low band gap energies are an efficient way [22, 23]. Excellent optical absorbance and perfect band gap energy of the semiconductors based on metal sulfide, chalcogenide and selenide including CdS [24, 25], CdSe [26], CuInS [27], Bi_2_S_3_ [28]and Ag_2_S [29] can be combined with ZNRs to produce various heterojunctions. However, the photoelectrodes made of heterogeneous structures of different metal-sulfide or chalcogenide-based semiconductors together with the ZNRs or arrays generally require some chemical reagents to consume the created holes [26], thus increasing the practical applications’ viability.

The applications of the semiconductor heterojunctions based on the metals' sulfide or selenide are limited mainly due to their complicated production processes and the need of costly reagents [30]. The low energy band gap MOSCs like Fe_2_O_3_ do not have the same band edge position as ZnO, resulting in a type-II band structure [31]. On top, they are photoconductive, nontoxic, and very stable. The low energy band gap semiconductor like In_2_S_3_ became promising due to the possibility of forming the heterojunctions with the ZNRs [32]. To achieve an efficient PEC for water splitting, the electro-deposition [33, 34] and successive ionic layer absorption and reaction (SILAR) [17] technique was used to make diverse In_2_S_3_/ZnO NWs. Strothkamper et al. [35] used the spray-ions-layer gas reaction method to synthesize various In_2_S_3_/ZnO NRs wherein the main aim was to determine their ability for the carriers’ separation and transport. Ming Li et al. [36] modified the electronic structure properties of ZnO by In_2_S_3_ nanosheet arrays using the atomic layer deposition method. Compared to the abovementioned methods for the In_2_S_3_/ZnO heterostructures synthesis, the SILAR approach is easier for producing the In_2_S_3_/ZnO core-shell heterostructures nanorod arrays (IZCSHNRAs) [37].

Based on the aforementioned factors, we used the SILAR approach to prepare some new types of IZCSHNRAs. The obtained samples were characterized using various techniques to determine the feasibility of enhancing their photoconversion performance (implemented as electrode in the PECs). The impact of varying SILAR cycles on the structures, morphologies, optical traits and photoconversion efficiency of the proposed IZCSHNRAs were evaluated. The electronic band structures of both ZnO and In_2_S_3_ was altered, enabling the alignment of the Type-II band and thus facilitating the photogenerated carriers’ effective transmission and separation. In addition, the feasibility of fabricating inexpensive and high quality IZCSHNRAs in a rapid way using the SILAR method with a small range of cycles and low concentration of indium chloride (cationic) as well as sodium sulfide (anionic) was demonstrated. The obtained results were analyzed, discussed and compared with other existing state-of-the-art literature reports (Table 1), indicating the outperforming nature of the proposed samples.

**Table 1:** Comparison of SILAR method of samples preparation with others techniques.

No	Concentration (M)		Immersion time (s)			Number of SILAR cycles	Ref.
	InCl_3_	Na_2_S	InCl_3_	Na_2_S	Rinsing		
1	0.1	0.05	40	40	50	100	[59]
2	In_2_(SO_4_)_3_ 0.08	0.05	25	25	-	55	[60]
3	0.04	0.1	20	20	15	50	[61]
4	0.01	0.01	6	6	2	100–300	[62]
5	0.01	0.01	-	-	-	50, 60, 80, 100, 150, 200	[63]
6	0.05	0.03	30	30	30	10,20,30,40,50	[64]
7	0.05	0.1	20	15	10	20	[65]
8	0.05	0.03	30	30	30	10,15,20	[66]
**9**	**0.001**	**0.03**	**12**	**12**	**30**	**2,4,6,8,10**	**Present Work**

## 2 Experimental methods

### 2.1 Preparation of samples

In this work, analytical grade chemical reagents (without further purification) and deionized water (DIW) were used to prepare all aqueous solutions and final products.

### 2.2 Synthesis of ZNRAs

First, Sn-doped polycrystalline In_2_O_3_ (called indium tin oxide or ITO of resistance of 10 U/sq) as substrates were ultrasonically cleaned successively for 15 min at 25 °C using acetone, 2-propanol and DIW. Then, all substrates were thoroughly rinsed in DIW to remove the impurities and activate the surface. The deposition of the ZnO nanoparticles seed layer onto ITO substrates was accomplished via the sol-gel unified spin coating using a precursor mixture (0.1 M) made of zinc acetate dihydrate [(CH_3_COO)_2_Zn._2_H_2_O)], ethanol (10 mL) and diethanolamine [CH_2_(OH)CH_2_)]_2_NH of 0.1 M followed by annealing at 400 °C for 1 h at a rate of 2 °C per minute. The conventional hydrothermal method (in water bath at 90 °C for 8 h) was used to grow ZNRAs. Next, the ZnO seed layer-coated ITO substrate was immersed in a sealed glass container containing [Zn(NO_3_)_2_.6H_2_O] and hexamethylenetetramine (HMTA [C_6_H_12_N_4_]) each of 40 mM. Afterward, the coated samples were rinsed using DIW before being dried by an air-blown gun. Finally, the ZNRAs-coated glass substrates were annealed in a furnace (Carbolite CWF 1200, UK) at 350 °C for 1 h at a heating rate of 2 °C per minute.

### 2.3 Synthesis of IZCSHNRAs

**Figure 1:**
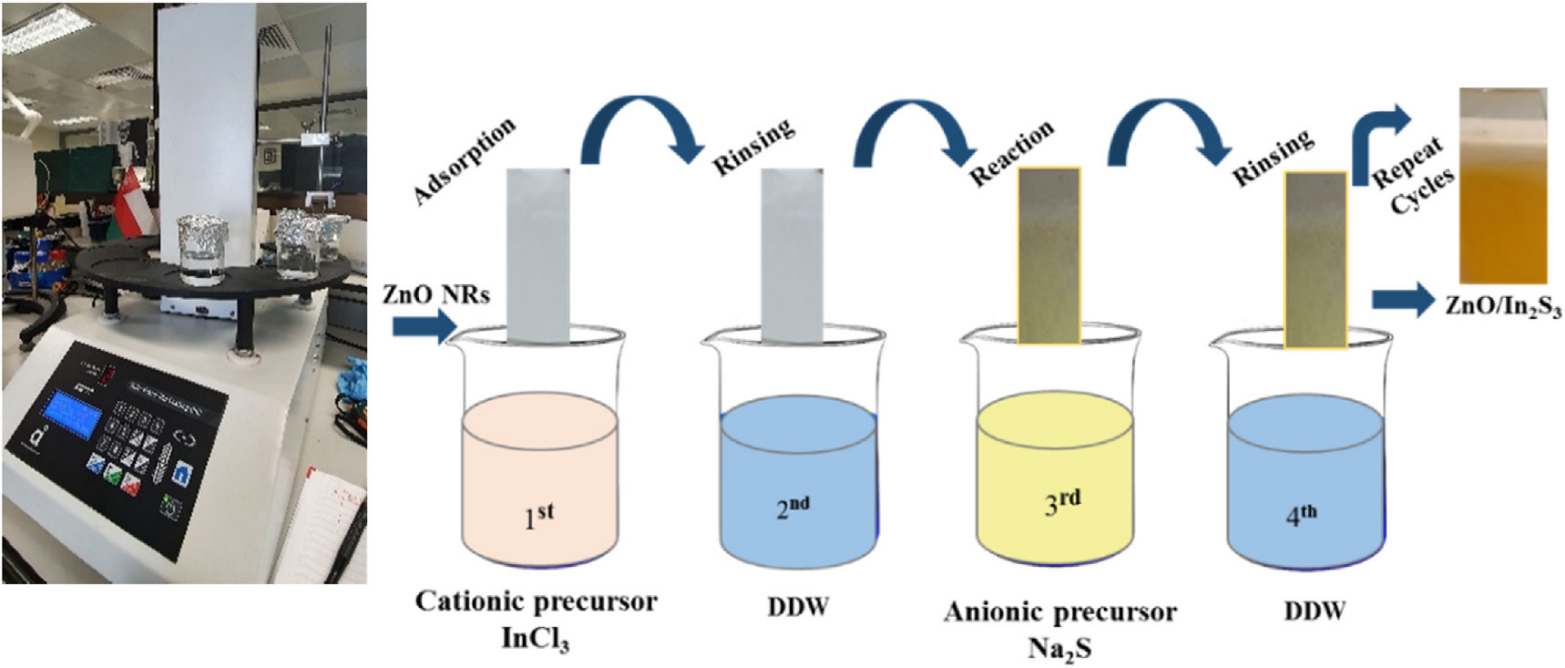
Scheme of SILAR approach for IZCSHNRAs synthesis and the photograph of Xdip-MV1 Apex Multi Vessel SILAR Coating instrument.

The as-deposited ZNRAs were used as a working electrode, subsequently immersed in a aqueous solution composed of indium chloride (InCl_3_, 0.001 M), sodium sulfide (Na_2_S, 0.03 M) (at controlled pH in the range of 8 and 9) and DIW before being subjected to the SILAR deposition technique (Multi-Vessel SILAR Coating Instrument Xdip-MV1, Apex) at various reaction cycles (2, 4, 6, 8 and 10). The obtained IZCSHNRAs were dried under nitrogen (N_2_) gas atmosphere followed by the thermal annealing at 200 °C for 30 min. The SILAR deposition system contained cationic precursor, ion exchange water, and anionic precursor. The equipment consisted of two 50 mL beakers containing the precursor solution and two rinsing vessels encircled on a circular tray. Each rinsing vessel was placed in between the beakers containing the cationic and anionic precursor solution. The Xdip-MV1 set-up combined a programmable robotic arm movement with both dip and SILAR coating process that included the dipping of a substrate into the multiple wells of the coating materials under controlled parameters for creating an alternate layer of the thin film on the substrate surface. In addition, one arm was used to connect the substrate vertically, enabling the arm to turn and slide tightly in a bearing. This state-of-the-art instrument is low-cost, sophisticated, versatile, and user-friendly. Figure 1 shows the SILAR cycle reaction scheme for the production of the proposed IZCSHNRAs. In this process, the obtained ZNRAs were first immersed in an aqueous solution of InCl_3_ for In^3+^ adsorption on the samples’ surface. Later, the samples were immersed in an aqueous solution of Na_2_S to react with In^3+^ and S_2_, producing the ISNPs on the NRAs surface. In short, a coating of In_2_S_3_ covered repeatedly the ZNRAs, thus creating the desired IZCSHNRAs.

### 2.4 Characterizations of samples

The physical, optical, and chemical properties of the IZCSHNRAs were measured using diverse techniques. The crystalline structures of the samples were verified using the X-ray diffraction measurement (XRD, Miniflex 600, Rigaku, Japan) in the angular scanning range (2θ) of 20°–80° at a resolution og 0.02°/s. Fourier transform infrared (FTIR) absorption spectra (PerkinElmer, Spectra One, USA) of the samples in the range of 400–4000 cm^−1^ at a resolution of 4 cm^−1^ with 40 scans were recorded to identify the chemical functional bonds**.** The samples morphologies and micro-structures were analyzed via the field emission scanning electron microscopy (FE-SEM: JSM-7800F, JEOL, Japan). A High resolution transmission electron microscope (HRTEM: Jeol JEM-2100F, Japan) attached to an Oxford links energy dispersive X-ray spectrometer (EDX) and interfaced with THE Gatan ENFINA EELS system was used to determine the morphology and chemical compositions of the samples. The X-ray photoelectron spectroscopy (XPS; Scienta Omicron, Germany) with Al-Kα line of energy 1486.6 eV as source (calibrated by C1s of energy 284.6 eV) was used to analyze the samples’ elemental composition and functional chemical groups or units. The UV-visible spectroscopy (PerkinElmer, Lambda 25, USA) in the range of 190–1100 nm was used to record the optical absorption spectra of the samples. The photoluminescence (PL) emission spectra of the samples were measured using a fluorescence spectrometer (LS 55, PerkinElmer, USA) with the excitation wavelength of 325 nm. All the characterizations were conducted at room temperature.

A PEC with 3-electrodes was designed to determine the photoconversion performance of the studied IZCSHNRAs wherein the IZCSHNRAs/ITO, platinum (Pt) wire, and silver/silver chloride (Ag/AgCl) served as the working, counter, and reference electrodes, respectively. A mixture of Na_2_SO_3_ (0.1 M) and Na_2_S of (0.1 M) of pH value 13 was used as the electrolyte. The linear sweep voltammetry (LSV) technique was used (Gamry Instrument framework interface with 1000 E Potentiostat/Galvanostat/ZRA) to measure the photocurrent density of the PEC with photoanode made from the proposed IZCSHNRAs. The potential was ranged from -1.2 to +1.2 V versus Ag/AgCl (scan rate of 20 mV s^-1^) in the presence of Fully Reflective Solar Simulator (SS 1.6 kW Sciencetech, Canada) operated at a power of 1600 W. A xenon lamp (Newport system) equipped with the filter of AM 1.5 G was used to simulate the atmospheric and terrestrial states of the solar irradiation. The intensity of the lamp output was matched with the solar irradiance spectra following the standard of ASTM G173-03 (2012). The light from the xenon lamp was focused on the quartz reaction cell over an area of (1 cm × 1 cm) of the working electrode placed at a separation of 16 cm. An irradiance of 100 mW/cm^2^ equivalent to one sun lighting was used wherein the light source was manually chopped at regular time interval. The photoconversion efficiency (η %) measurements were conducted at the ambient atmospheric conditions and repeated thrice to guarantee the reproducibility of the results with good statistical accuracy. Usually, an external voltage was required in the PEC water splitting system and the electrical energy was subtracted from the energy gain. The values of η were evaluated using Eq. (1):

(1) $$\text{Photoconversion ​efficiency ​}\left(\text{η}\right)=\frac{J_{ph}\left(1.23-V_{app}\right)}{P_{in}}\;$$

where *P*_*in*_ is the incident light irradiance (100 mWcm^−2^, AM 1.5G), *J*_*P*_ (*J*_*P*_ = *J*_*L*_ –*J*_*D*_) denotes the achieved photocurrent density (in mAcm^−2^) under the external applied voltage (*V*_*app*_). The thermodynamic water splitting potential slandered the reversible redox potential of H_2_O electrolysis in accordance with the standard H electrodes (NHE) is 1.23 V.

## 3 Results and discussion

### 3.1 Structural analysis

**Figure 2:**
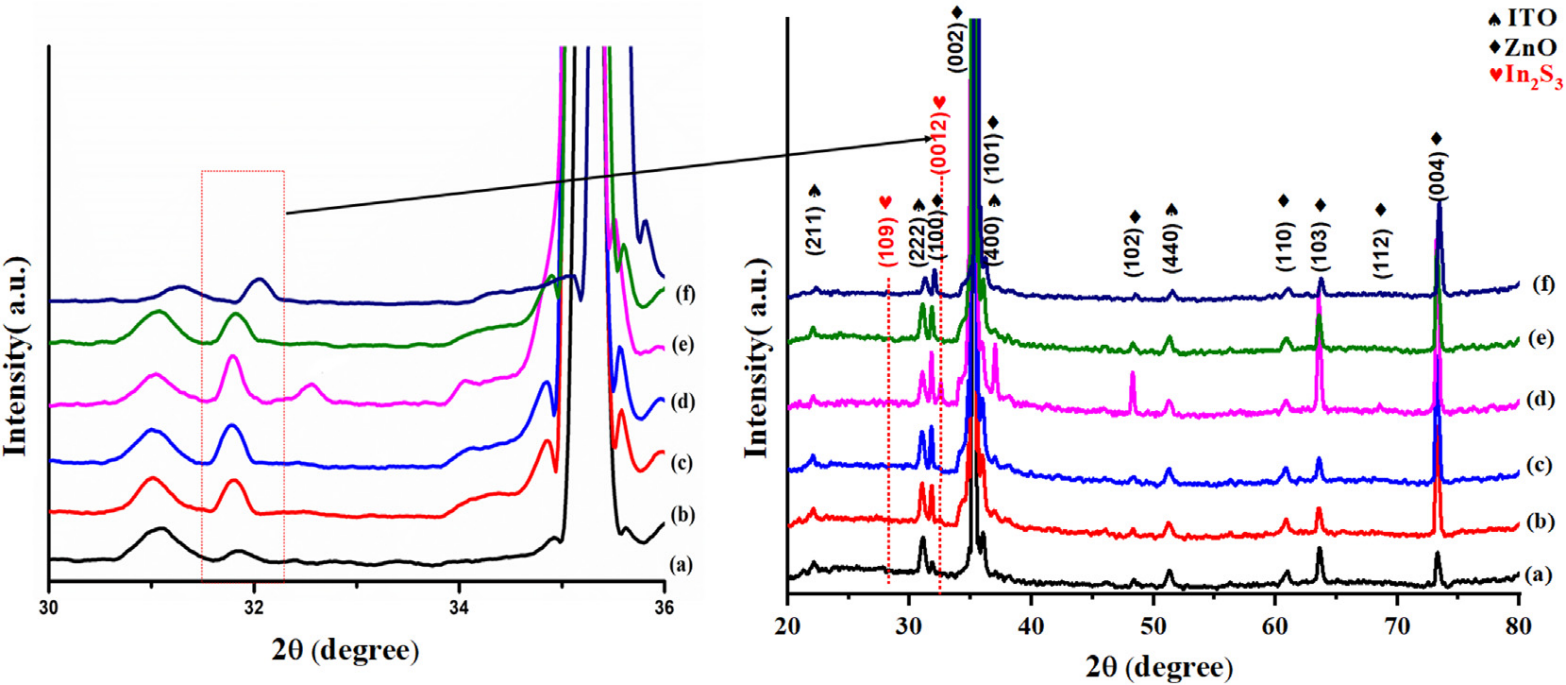
XRD patterns of the proposed ZNRAs, IZCSHNRAs-C2, IZCSHNRAs-C4, IZCSHNRAs-C6, IZCSHNRAs-C8, and IZCSHNRAs-C10. The inset represents the magnified peaks centered at 30^o^ to 36^o^.

Figure 2 displays the XRD profiles of the pure ZNRAs and IZCSHNRAs prepared using various deposition cycles. The ZNRAs exhibited a pure crystalline phase with wurtzite structures (matched with the JCPDS card number 04-008-7114). The diffraction peaks corresponding to the (100) (002) (101) (102) (110) (103) (112) and (004) planes were positioned at 2θ = 31.78°, 34.84°, 36.13°, 48.21°, 57.35°, 63.57°, 69.13°, and 73.18°, respectively. These observations were in good agreement with other reports [17, 33]. After placing the In_2_S_3_ coating over the ZNRAs, the presence of the predominant (002) XRD peak clearly showed that ZNRAs could grow with their c-axis orientation normal to the ITO surface. After the deposition of In_2_S_3_, two new peaks were observed in addition to the diffraction peaks from the hexagonal ZNRAs. These two broad peaks centered at 28.15° and 32.61° corresponded to the (109) and (0012) diffraction planes of the tetragonal crystal structure of β-In_2_S_3_ (tallied with the JCPDS card number 00-025-0390). These results agreed well with other reported findings [17, 33, 34]. The formation of the tetragonal In_2_S_3_ can be ascribed to the stability of this phase at room temperature [38]. The low intensity of the peaks can be related to the poor crystallinity of the shell formed over the ZNRAs. In addition, the intensity of the characteristic XRD peaks of IZCSHNRAs was first increased with the increase of the deposition cycles from 2 to 6, and then decreased up to 10 cycles. This clearly indicated that the amount of the deposited ISNPs was increased with the deposition cycles from 1 to 6. It is further argued that the deposition cycles of the SILAR process was correlated to the (i) growth mechanism of the In_2_S_3_ shell and (ii) improved crystallinity due to the structural and morphological modifications of the In_2_S_3_ shell. In short, the deposition cycles were responsible for the increase of the mean crystallite size and surface roughness of the samples [33, 39, 40, 41]. The XRD data analyses confirmed the successful deposition of the In_2_S_3_ shell on the ZNRAs surface, thus forming the core-shell NRAs. It was concluded that the SILAR deposition method with varying cycles is an effective way to generate the customized IZCSHNRAs.

The average crystallite size of the In_2_S_3_ shell was calculated following the Debye Scherrer Eq. (2) [42] wherein the prominent XD peak corresponding to the (0012) growth plane was considered (Table 2):

(2) $$D=\frac{K\lambda }{\beta Cos\theta }$$

where *λ* (*≈*1.5406 Å) is the wavelength of Cu-Kα radiation, *β* is the full-width at half maximum (FWHM) of the intense diffraction peak, *θ* is the Bragg diffraction angle, and *K* is a constant equal to 0.94.

**Table 2:** Equivalent circuit parameters for IZCSHNRAs-based electrodes prepared with various deposition cycles.

Photoelectrodes	Crystalline size D (nm)	E_g_ (eV)	J_ph_ (mA/cm^2^)	η (%)
ZNRAs	21.95	3.23	0.63	0.46
IZCSHNRAs-C2	28.35	2.47	0.77	0.56
IZCSHNRAs-C4	27.54	2.44	0.84	0.61
IZCSHNRAs-C6	23.75	2.32	1.05	0.77
IZCSHNRAs-C8	25.48	2.37	0.92	0.68
IZCSHNRAs-C10	36.06	2.40	0.90	0.66

The average crystallite size in the In_2_S_3_ shells (Table 2) were decreased from 28.35 nm to 23.75 nm with the increase of the deposition cycles deposition from 2 to 6. The observed reduction in the crystallite size can be attributed to the weakening of the van der Waals interactions between different crystallites, leading to an improvement in the samples crystallinity. Similar results were obtained by Braiek et al. [33] and Chunmei et al. [39]. At higher number of the deposition cycles like 8 and 10 the corresponding average crystallite sizes were slightly increased to 25.48 and 36.06 nm, which can be ascribed to the increase of the deposited particles coalescence because of the substrate immersion in the solution for prolonged duration [43].

**Figure 3:**
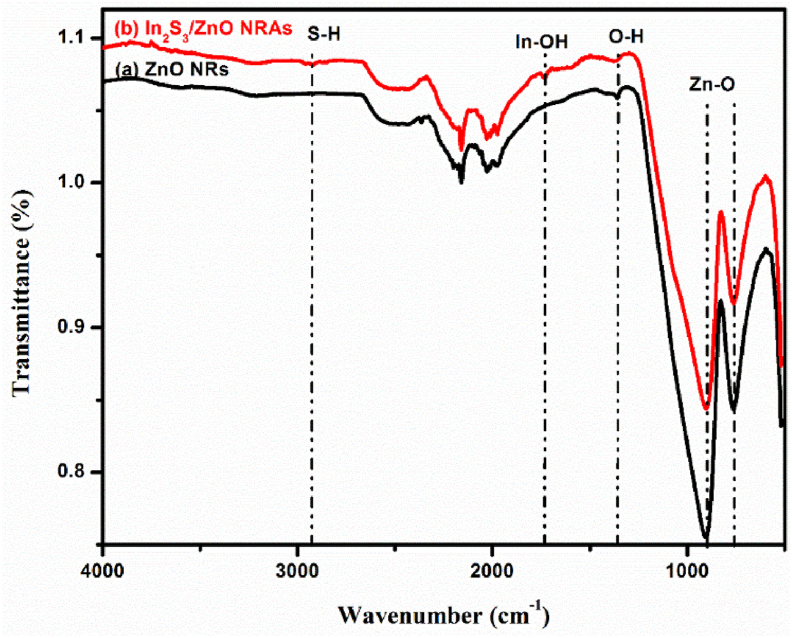
IR transmittance spectra of the ZNRAs and IZCSHNRAs-C6.

Figure 3 illustrates the FTIR spectra of the pure ZNRAs and IZCSHNRAs (optimum one prepared at 6 cycles). Table 3 shows the FTIR peak assignments of the studied samples, displaying the corresponding functional chemical bonds. The broad and asymmetric band at 1355 cm^−1^ was assigned to the stretching vibration of the hydroxyl (O–H) group in the sample. The observed peak related to the O–H bending modes in the IR region was consistent with other report [44]. The peak at 2935 cm^−1^ corresponded to the S–H vibration mode of thiol and was observed only in the IZCSHNRAs. The IR band at 1739 cm^−1^ was assigned to the In–OH deformation modes [45, 46]. For both samples, the IR peaks at 907 cm^−1^ and 760 cm^−1^ were assigned to the Zn–O bond vibration [47].

**Table 3:** EDX results of the IZCSHNRAs.

Fitting Coefficient:			0.6952			
Element	(keV)	Mass%	Counts	Error%	Atom%	K
O K	0.525	29.73	4840.43	0.03	64.83	0.7686
S K	2.307	2.51	635.17	0.48	2.73	0.494
Zn K (Ref.)	8.63	51.6	6456.94	0.08	27.52	1
In L	3.286	16.17	1253.07	0.3	4.91	1.6146
Total		100			100	

**Figure 4:**
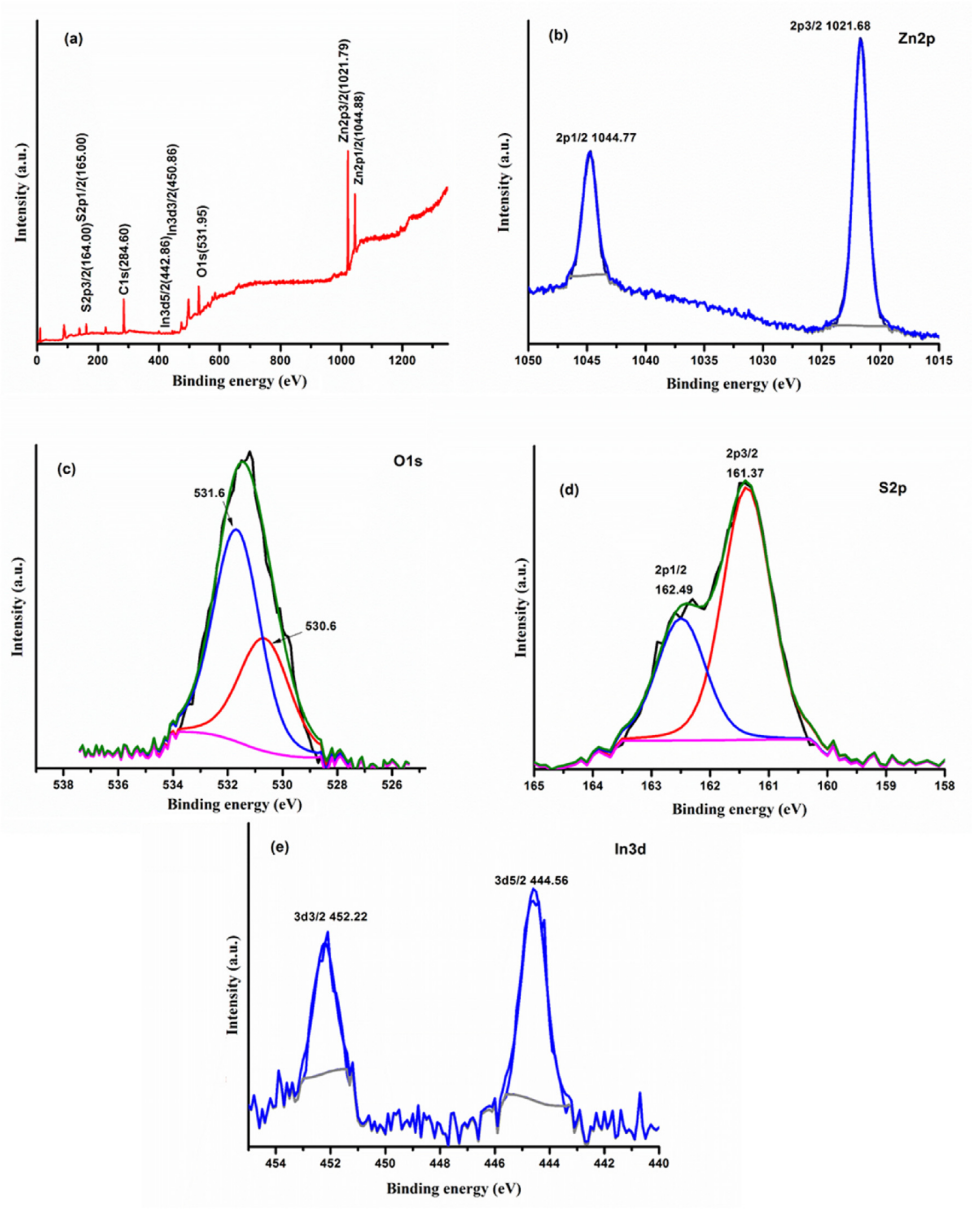
XPS spectra of the (a) IZCSHNRAs-C6 (b) Zn 2p (c) O 1s (d) S 2p and (e) In 3d.

Figure 4(a-e) depicts the XPS spectrum of the samples (prepared at 6 cycles), revealing the right chemical composition and states. The IZCSHNRAs (Figure 4a) exhibited the peaks corresponding to the chemical elements Zn, O, S and In. Figure 4b–e displays the binding energies of Zn, O, S, and In, respectively. The peak of Zn 2p (Figure 4b) centered at 1021.86 and 1044.77 eV verified the presence of Zn^2+^ in the IZCSHNRAs. The binding energy peaks of O 1s (Figure 4c) was divided into two regions α and β positioned at 530.6 and 531.6 eV, respectively which were due to the hydroxyl ions at the surface and oxygen in the lattice sites of ZnO [48]. The binding energy peaks of S 2p at 161.37 and 162.49 eV (Figure 4d) clearly demonstrated the presence of S in the IZCSHNRAs. The observed binding energy peaks of In 3d (Figure 4e) at 444.56 and 452.22 eV in the current study are in conformity with other reports [17, 49]. In short, the present findings confirmed that the ISNPs were successfully grown on ZNRAs using the SILAR deposition technique.

### 3.2 Morphological analyses

**Figure 5:**
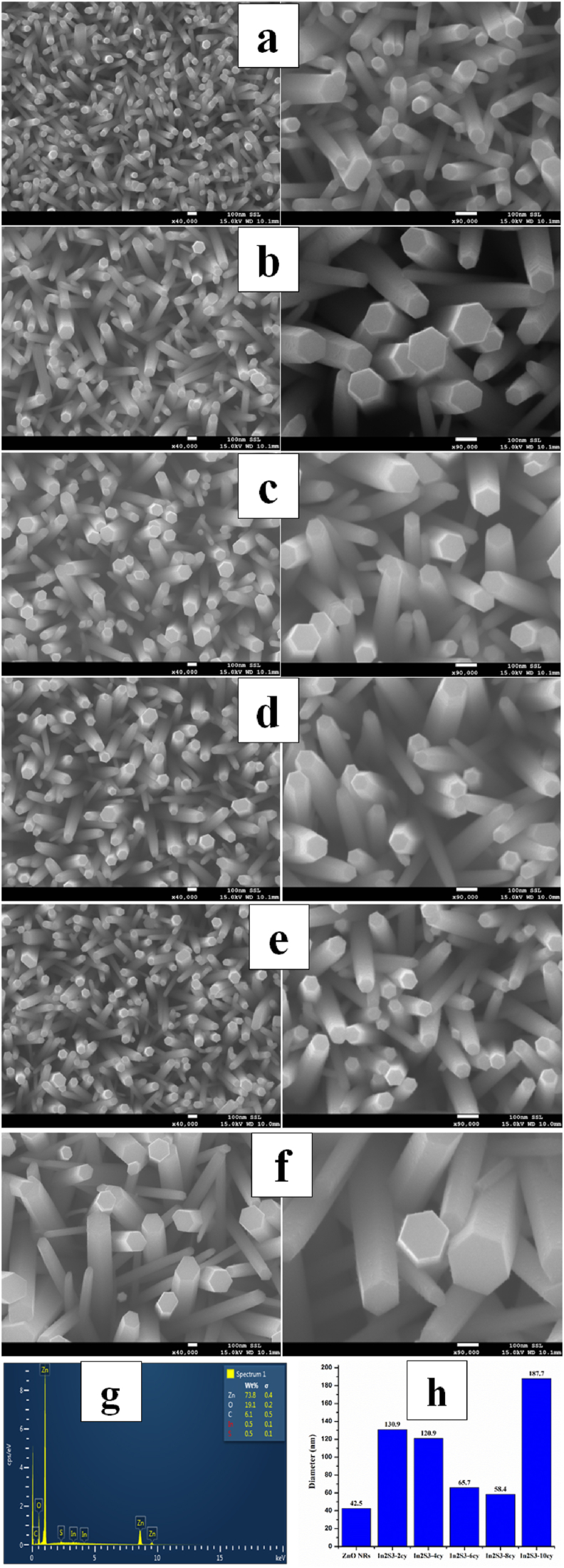
FESEM images (top view) of the (a) bare ZNRs, (b) IZCSHNRAs-C2, (c) IZCSHNRAs-C4 (d) IZCSHNRAs-C6, (e) IZCSHNRAs-C8, (f) IZCSHNRAs-C10, (g) EDX spectra and (h) NRAs diameter.

Figure 5(a-h) presents the effects of various deposition cycles on the FESEM surface morphology of the IZCSHNRAs and pure ZNRAs. The FESEM micrographs revealed the nucleation of dense and vertically aligned hexagonal ZNRAs (Figure 5a) with homogeneous distribution, covering the entire substrate surface. Figure 5b-f shows the FESEM images of the respective core-shell samples obtained using the In_2_S_3_ shells at 2–10 deposition cycles. These results clearly showed the formation of a continuous In_2_S_3_ shell around the ZNRAs supported on a transparent conductive substrate. In addition, as the number of deposition cycles was increased up to 8, the average diameter of the In_2_S_3_/ZnO core-shell was reduced (calculated by ImageJ software), thereafter the diameters were increased significantly to approximately 187 nm at 10 cycles (Figure 5h). Compared to the pure ZNRAs, the upper diameter of the IZCSHNRAs was thinner than the diameter at middle portion. This was mainly due to the partial aggregations of In_2_S_3_ on top of the ZNRAs wherein the diameter of the IZCSHNRAs became thicker. The length of the IZCSHNRAs obtained from the TEM image was 1.25 μm Figure 5g shows the EDX spectra of the pure ZNRAs and IZCSHNRAs, reconfirming the formation of the pure In_2_S_3_ shell over the NRAs and appropriate elemental composition.

**Figure 6:**
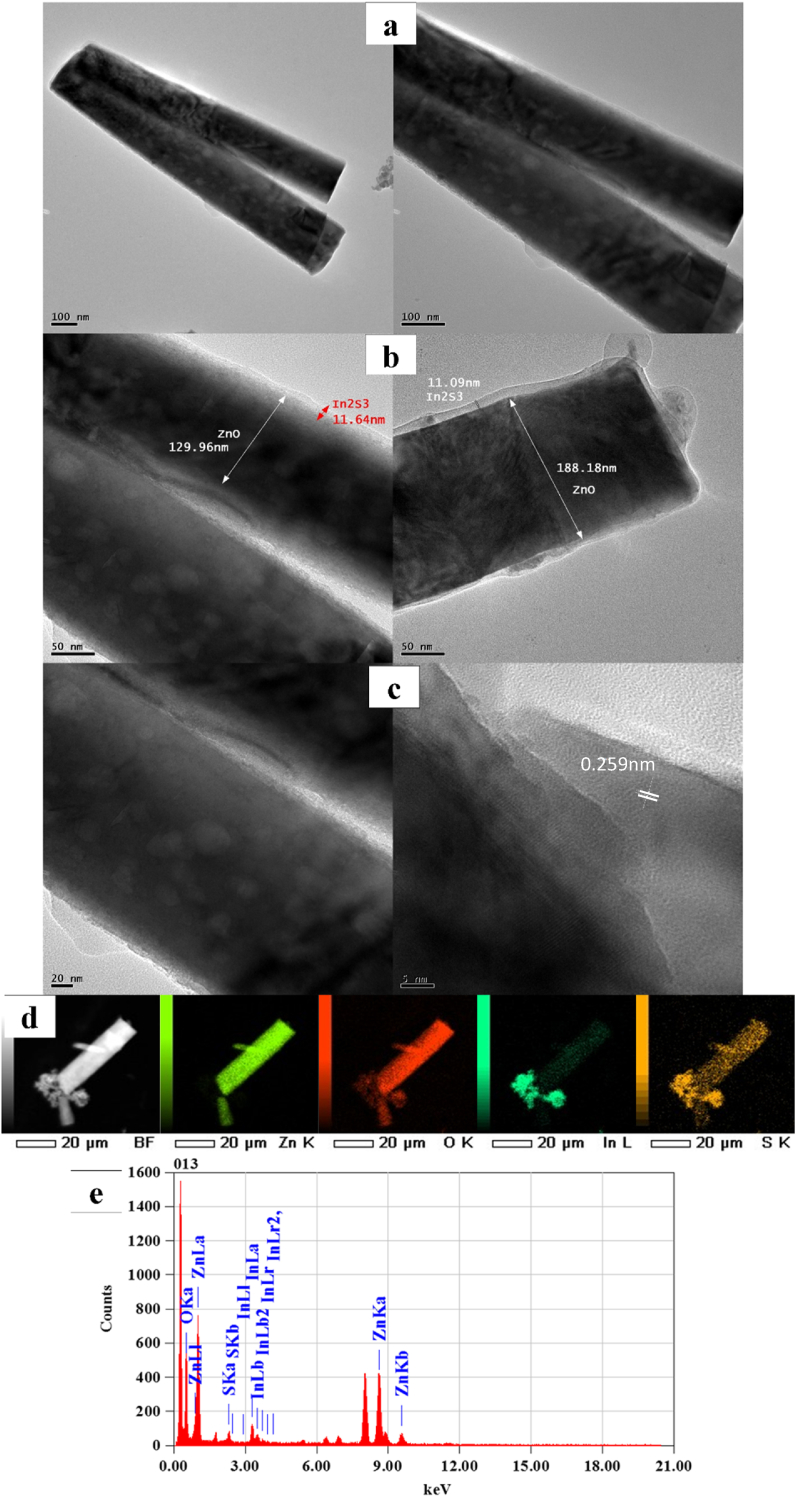
(a–c) HRTEM images and TEM images of the IZCSHNRAs-C6, (d) EDX elemental maps of Zn, O, S, and In and (e) EDX spectra.

The TEM images (Figure 6(a-e)) revealed the nucleation of an uniform envelop of the ZNRAs within the In_2_S_3_ shells of varying thicknesses. When the number of SILAR cycles reached to 6, the thickness of the In_2_S_3_ layer was approximately 11 nm and the fringe spacing of the In_2_S_3_ layer was estimated to be 0.259 nm (Figure 6e) which corresponded to the In_2_S_3_ (0012) growth plane, thus supporting the XRD results (Figure 2). Figure 6d displays the EDX elemental maps of the IZCSHNRAs, validating the presence of the elements Zn, O, S, and In with their uniform dispersion throughout the sample. In addition, the elements Zn and O were diffused in the sulfur core and indium was diffused into the shell. Figure 6e shows the EDX spectral analysis of the IZCSHNRAs, wherein the presence of various chemical elements (Table 4) indicated the expected atomic percentages of oxygen, zinc, indium, and sulfur. Briefly, the achieved results suggested the prospect of the SILAR approach for the successful deposition of the IZCSHNRAs with desirable characteristics needed for practical applications.

**Table 4:** Transmittance bands position and assignments of the obtained ZNRAs and IZCSHNRAs.

ZNRAs	IZCSHNRAs	IR band assignment
1355	1355	O–H stretching
-	2935	S–H stretching
-	1739	In–OH
907	907	Zn–O
760	760	Zn–O

### 3.3 Optical characteristics

**Figure 7:**
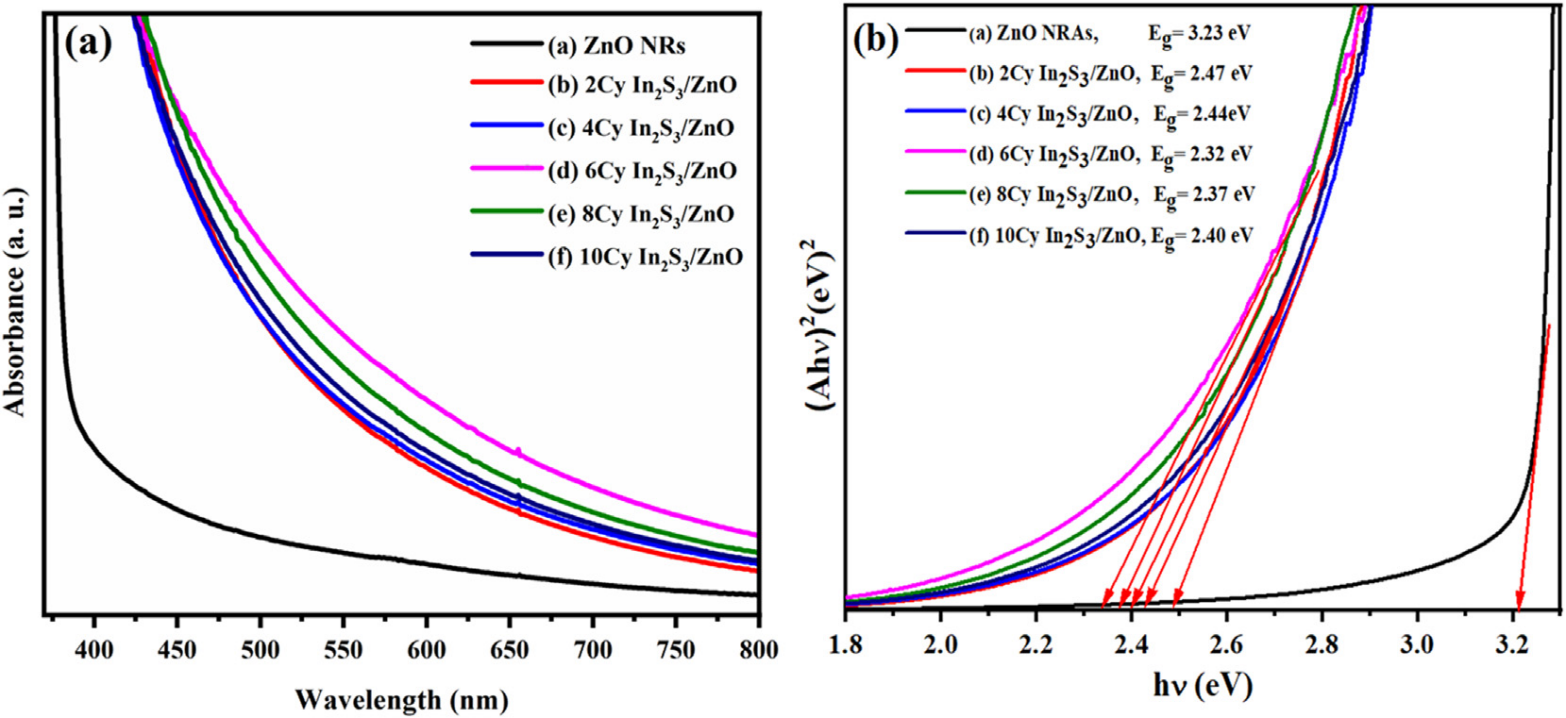
(a) UV–Vis spectra and (b) (*αhν*)^2^ versus photon energy plot of the pure ZNRAs and IZCSHNRAs prepared using various deposition cycles.

Figure 7a shows the optical absorption spectra of the ZNRAs and IZCSHNRAs synthesized using various deposition cycles wherein the UV absorption-edge was observed at 400 nm. The absorption spectra consisted of various significant peaks in the range of 500–600 nm. The visible absorbance of the In_2_S_3_ showed enhancement, indicating the light harvesting potential of the proposed samples. Figure 7b shows the Tauc plot ((*αhν*)^2^ against photon energy (*hν*)) of the ZNRAs and IZCSHNRAs synthesized at different SILAR deposition cycles. The values of the energy band gap of the samples were estimated via Eq. (3) [33]:

(3) $$\left(\alpha hv\right)=A{\left(hv-Eg\right)}^{n}$$

where *α* is the absorption coefficient, *h* is the Planck's constant, *ν* is the frequency of the incident light, *A* is a constant specific to the material, *E*_g_ is the band gap energy of the sample under study and *n* is an index that depends on the nature of the transition across the optical band gap of the material wherein *n* = 2 for direct band gap material like ZnO and In_2_S_3_. The values of *E*_g_ for the pure ZNRAs and IZCSHNRAs were ≈3.23 eV and in the range of 2.47 to 2.32 eV, respectively. With the increase of the deposition cycles from 2 to 6, the values of *E*_g_ for the IZCSHNRAs were decreased, indicating an improved absorbance of the materials. However, with the increase of the deposition cycles from 8 to 10, the values of *E*_g_ for the IZCSHNRAs were increased from 2.37 and 2.40 eV, respectively. These results confirmed an appreciable visible photo-response of the materials because of low *E*_g_ and *α* values [50].

**Figure 8:**
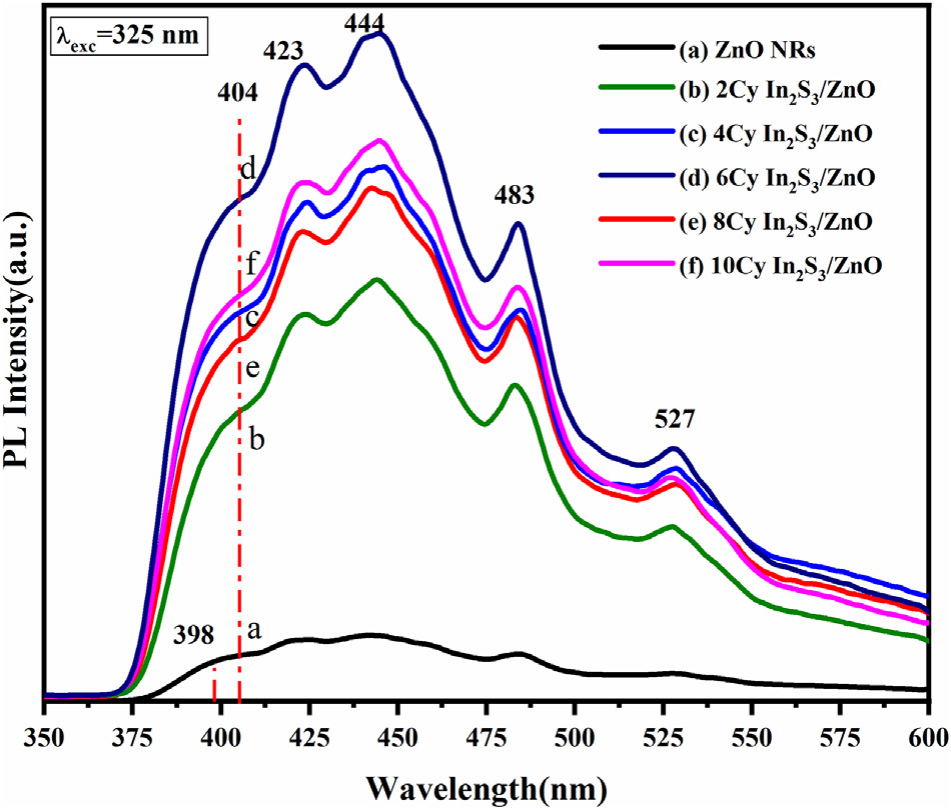
Room temperature PL spectra of the bare ZNRAs and IZCSHNRAs prepared using various deposition cycles.

Figure 8 shows the PL emission spectrum of the pure ZNRAs and IZCSHNRAs at an excitation wavelength of 325 nm. The PL data analyses provided the information about the quality and origin of defects into the materials including donors, acceptors, vacancies, and interstitials. The PL spectrum of the ZNRs (Figure 8a) revealed three emission bands and matched with the previous studies [41, 51, 52, 53] wherein a strong UV emission at 398 nm was evidenced. This observation was attributed to the near band edge (NBE) emission from ZnO. The observed strong blue peak in the range of 423–444 nm and a shoulder (green emission band) in the range of 483–527 nm was due to the presence of singly ionized oxygen vacancy of ZnO originated from the recombination of a photo-generated hole with the singly ionized charged state of the defect. In brief, all samples displayed a strong doubly-peaked emission band in the UV region positioned at 403 and 423 nm that can be attributed to the quantum confine effects in_2_S_3_ [54]. The observed weakly intense peak in the blue region (444 nm) and another in the green region (527 nm) may be due to the transition between the vacancies of indium and sulfur (V_In_-V_S_) [55]. The PL response of the IZCSHNRAs were appreciably different compared to the pure ZNRs, displaying the luminescence intensity enhancement accompanied by the red-shift (≈6 nm). This red-shift enabled an improvement in the optical absorbance of the ZNRAs, thus enhancing the current density when used them as electrodes. The lattice mismatch between the ZnO and In_2_S_3_ caused a strong electronic interaction, responsible for the observed red-shift in the PL spectra [56]. Furthermore, the visible peak intensities of the IZCSHNRAs were much higher than that of the pure ZNRAs, indicating the possibility of light re-absorption at the In_2_S_3_/ZnO interface. In fact, numerous researchers observed such decrease in the UV emission and explained such behavior in terms of the type-II interfacial transition mechanism between the valence and conduction band of the two semiconductors [41, 57, 58].

### 3.4 Photoconversion performance of IZCSHNRAs

**Figure 9:**
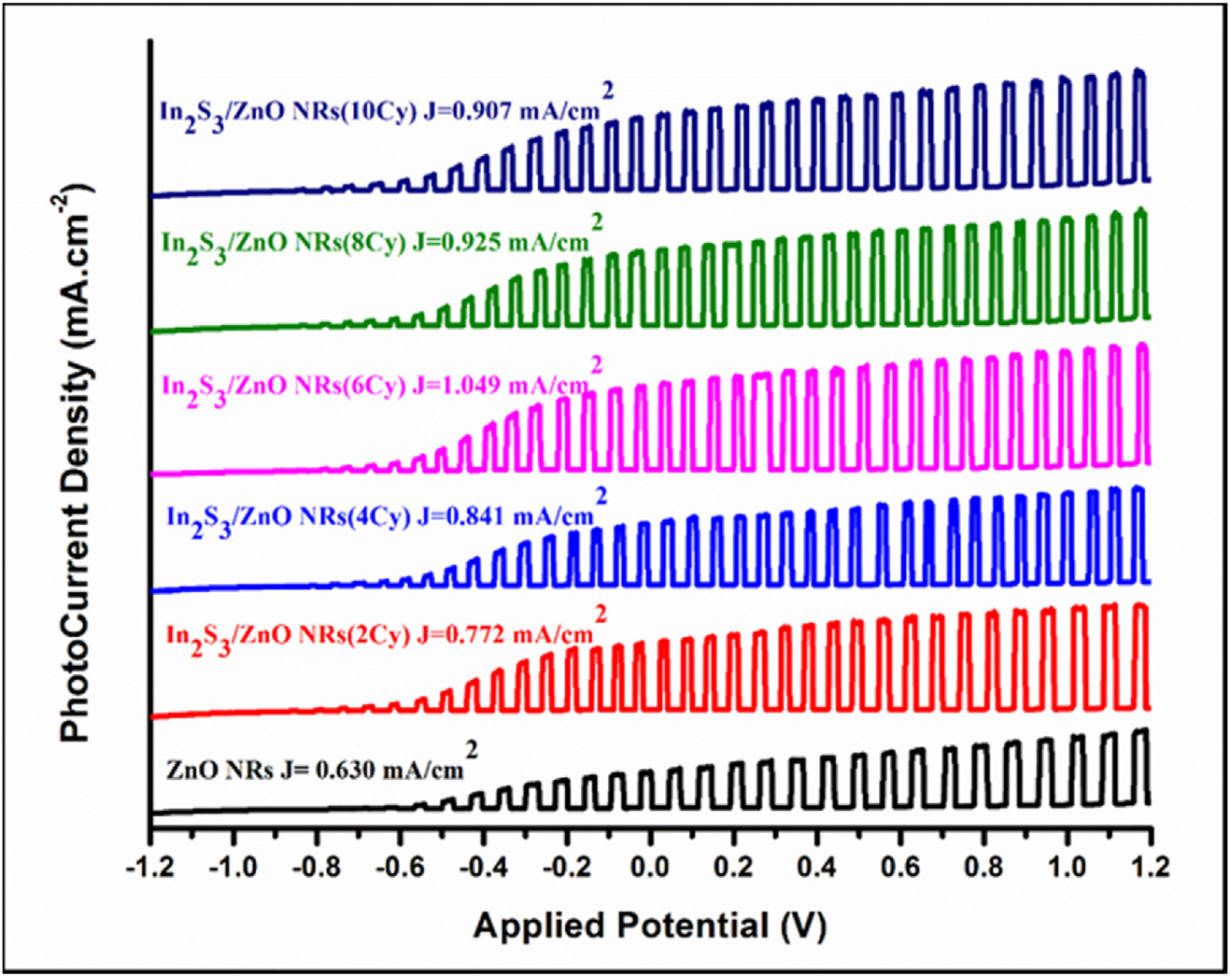
Linear sweep voltammograms of the bare ZNRAs, IZCSHNRAs-C2, IZCSHNRAs-C4, IZCSHNRAs-C6, IZCSHNRAs-C8, and IZCSHNRAs-C10 obtained with the applied potential of 0.5 V.

Figure 9 displays the linear sweep voltammograms of the bare ZNRAs, IZCSHNRAs-C2, IZCSHNRAs-C4, IZCSHNRAs-C6, IZCSHNRAs-C8, and IZCSHNRAs-C10 obtained using the applied potential of 0.5 V. The photoconversion performance of the designed PEC with the IZCSHNRAs-based electrode was evaluated. The photocurrent density (*J*_*ph*_) of the IZCSHNRAs measured at +0.5 V was first increased from 0.772 to 1.05 mAcm^−2^ with the increase of the deposition cycles from 2 to 6 and then decreased at higher deposition cycles (from 8 and 10). The values of *J*_*ph*_ for the IZCSHNRAs were higher than the one obtained with ZNRAs-electrode. Furthermore, the IZCSHNRAs-C6 exhibited the maximum *J*_*ph*_ of 1.05 mA/cm^2^ that was nearly two fold higher than the one achieved using the pure ZNRAs. When the number of the deposition cycles were below 8, the formation of the In_2_S_3_ layer was very thin thereby weakly promoting the separation of the photo-generated charge carriers, thus limiting the photocurrent enhancement. However, when the number of SILAR cycles was above 6 the deposited layer of the In_2_S_3_ was very thick wherein the presence of excess In_2_S_3_ could block the photo-generated carriers' injection into the ZNRAs and enhance the photo-generated carriers’ recombination, thus decreasing the photocurrent values.

**Figure 10:**
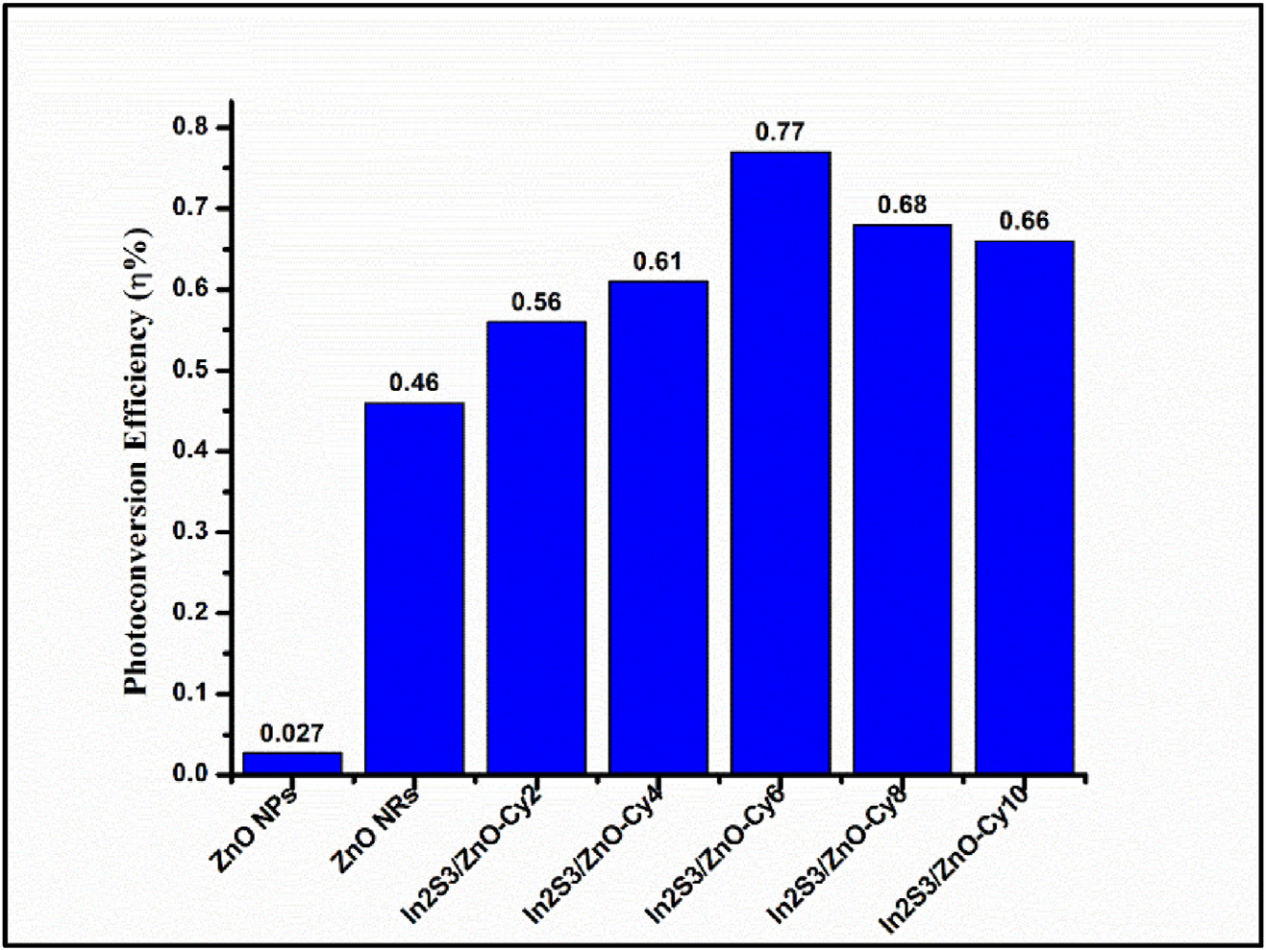
Photoconversion efficiency (η) of the ZNPs (seed layer), ZNRAs, IZCSHNRAs-C2, IZCSHNRAs-C4, IZCSHNRAs-C6, IZCSHNRAs-C8, and IZCSHNRAs-C10 obtained with the applied potential of 0.5 V.

Figure 10 illustrates the photoconversion efficiency (η) of the ZNPs (seed layer), ZNRAs, IZCSHNRAs-C2, IZCSHNRAs-C4, IZCSHNRAs-C6, IZCSHNRAs-C8, and IZCSHNRAs-C10 obtained using the applied potential of +0.5 V. The values of η obtained with +0.5 V were found to increase from 0.56 to 0.77 with the corresponding increase of the deposition cycles from 2 to 6. Further increase in the SILAR cycles caused a decrease in the values of η, indicating a reduction in the photocurrent density of the cell. The calculated value of η for the deposition cycles of 6 was much higher compared to the one obtained for the bare ZNRAs (0.46%). This disclosure can be ascribed to the significant absorption by the proposed material and excellent band alignment levels of the photo-anodes’ components. As the SILAR cycles were increased from 2 to 6, the absorption of the incident solar photons by the electrode was increased and more electron-hole pairs were formed, thus enhancing the values of both *J*_*ph*_ and η%. Furthermore, with the further increase of the number of SILAR cycles the absorption edge was red-shifted, inducing the generation of more electron-hole pairs and thus enhancing the values of both *J*_*ph*_ and η. It was argued that the deposited ZNRAs onto the In_2_S_3_ layer could efficiently enable the transport of the photogenerated electrons into the ZNRAs and gather the holes at the interface during the oxygen evolution reaction in PEC, thus efficiently separating the photogenerated electron-hole pairs and strongly suppressing the carriers' recombination. Essentially, the development of the IZCSHNRAs enabled the photogenerated electron-hole pairs separation, thus elongating the carriers’ lifetimes.

Table 2 displays the equivalent circuit parameters of the IZCSHNRAs electrodes prepared using various deposition cycles. It was further asserted that an increase in the number of SILAR cycles beyond 6 could cause an excessive amount of In_2_S_3_ in the coated layer that might have blocked the spaces between the ZNRs required for an efficient electron transport, inducing the promotion of the recombination processes of the carriers than the generation and thus leading to the lowering of *J*_*ph*_ and η values of the photoanode. In a nutshell, the observed improvement in the values of both *J*_*ph*_ and η with the increase of the number of SILAR cycles clearly affirmed that the PEC performance can be customized by controlling the photo-generated electron-hole pairs recombination rate wherein the In_2_S_3_ layer thickness, band alignments, formation of the core-shell NRAs that act as the recombination center together with the structure, morphology and optical properties of the IZCSHNRAs electrodes play a significant role.

## 4 Conclusion

Six PEC electrodes based on the proposed ZNRAs and IZCSHNRAs prepared using various SILAR deposition cycles (2, 4, 6, 8, and 10) were designed and their photoconversion performances were evaluated. It was shown that the structure, morphology, and optical properties of the IZCSHNRAs can be tailored by controlling the number of the SILAR deposition cycles. The layer thickness of the In_2_S_3_, band alignments, and formation of the core-shell ZNRAs heterostructures were demonstrated to be very sensitive to the deposition cycles variation. The obtained significant improvement in both *J*_*ph*_ and η values of the designed PEC was explained in terms of various mechanisms wherein the improvement of the overall characteristics of the IZCSHNRAs played a vital role. The IZCSHNRAs-C6 sample displayed the maximum photocurrent density of 1.05 mAcm^−2^ and photoconversion efficiency of 0.77% which was two times more than the one obtained for the bare ZNRAs (0.46%) and 28 times above that of ZNPs seed layer (0.027%). In short, the proposed systematic strategy for the fabrication of the IZCSHNRAs-based photoelectrodes using the economic SILAR approach may constitute a basis for the progress of high performance PECs required for clean energy production, contributing to sustainable development.

## Declarations

### Author contribution statement

Mohammed Rashid Almamari: Conceived and designed the experiments; Performed the experiments; Analyzed and interpreted the data; Contributed reagents, materials, analysis tools or data; Wrote the paper.

Naser M. Ahmed, Araa Mebdir Holi: Conceived and designed the experiments; Analyzed and interpreted the data.

F. K. Yam, Mohammed Z. Al-Abri, Basma A. El-Badry, M. A. Ibrahem: Contributed reagents, materials, analysis tools or data.

M.A. Almessiere: Analyzed and interpreted the data; Wrote the paper.

Osamah A. Aldaghri, Khalid Hassan Ibnaouf: Contributed reagents, materials, analysis tools or data; Analyzed and interpreted the data.

### Funding statement

This study was supported by Scientific Research at the Imam Mohammad Ibn Saud Islamic University [RG-21-09-46].

### Data availability statement

Data included in article/supp. material/referenced in article.

### Declaration of interest's statement

The authors declare no conflict of interest.

### Additional information

No additional information is available for this paper.
